# *Bifidobacterium animalis* subsp. *lactis* MN-Gup protects mice against gut microbiota-related obesity and endotoxemia induced by a high fat diet

**DOI:** 10.3389/fnut.2022.992947

**Published:** 2022-11-02

**Authors:** Xiaokang Niu, Nana Zhang, Shusen Li, Ning Li, Ran Wang, Qi Zhang, Jingjing He, Erna Sun, Xiaohong Kang, Jing Zhan

**Affiliations:** ^1^Beijing Laboratory of Food Quality and Safety, College of Food Science and Nutritional Engineering, China Agricultural University, Beijing, China; ^2^Key Laboratory of Precision Nutrition and Food Quality, Department of Nutrition and Health, China Agricultural University, Beijing, China; ^3^Mengniu Hi-Tech Dairy Product Beijing Co., Ltd., Beijing, China; ^4^R&D Center, Inner Mongolia Mengniu Dairy (Group) Co. Ltd., Huhhot, China; ^5^Key Laboratory of Functional Dairy, Department of Nutrition and Health, China Agricultural University, Beijing, China

**Keywords:** *Bifidobacterium animalis* subsp. lactis MN-Gup, obesity, dyslipidemia, endotoxemia, gut microbiota

## Abstract

Obesity has become a public health concern due to its global prevalence and high risk of complications such as endotoxemia. Given the important role of gut microbiota in obesity, probiotics targeting gut microbiota have been developed and applied to alleviate obesity. However, most studies focused on the effects of probiotics on pre-existing obesity, and the preventive effects of probiotics against obesity were rarely studied. This study aimed to investigate the preventive effects of *Bifidobacterium animalis* subsp. *lactis* MN-Gup (MN-Gup) and fermented milk containing MN-Gup against high fat diet (HFD)-induced obesity and endotoxemia in C57BL/6J mice. The results showed that MN-Gup, especially the high dose of MN-Gup (1 × 10^10^CFU/kg b.w.), could significantly protect mice against HFD-induced body weight gain, increased fat percentage, dyslipidemia, and increased lipopolysaccharides (LPS). Fermented milk containing MN-Gup had better preventive effects on fat percentage and dyslipidemia than fermented milk without MN-Gup, but its overall performance was less effective than MN-Gup. Furthermore, MN-Gup and fermented milk containing MN-Gup could alter HFD-affected gut microbiota and regulate obesity- or endotoxemia-correlated bacteria, which may contribute to the prevention of obesity and endotoxemia. This study revealed that MN-Gup could reduce obesity and endotoxemia under HFD, thereby providing a potential application of MN-Gup in preventing obesity.

## Introduction

Obesity has been recognized as a chronic disease characterized by excessive accumulation of fat. In the past decades, obesity prevalence has increased and become an important factor of disability and death in many parts of the world (1, 2), posing a growing threat to public health worldwide. Obesity has negative effects on the quality of life and work efficiency. An obese person is reported to spend an additional 1,901 dollars on illness per year (2). Undoubtedly, obesity increases the huge financial burden on families and society.

Obesity is associated with increased risks of diseases including diabetes, hyperlipidemia, high blood pressure, heart disease, etc. (3, 4). In addition, obesity is often accompanied by endotoxemia characterized by the excessive accumulation of lipopolysaccharides (LPS) in the blood circulatory system (5). LPS is a component of the cell walls of Gram-negative bacteria (6), and its presence to a limited extent in the intestinal lumen may not cause harmful effects. LPS could cross the gut barrier and transfer into the blood circulatory system due to gut barrier impairment and gut microbiota dysbiosis, further triggering toll-like receptor 4 (TLR4)-mediated immune responses that lead to inflammation (7). Thus, it is of great significance to study the strategies to prevent and alleviate obesity.

In recent years, gut microbiota is found to play an important role in obesity, and dietary interventions for obesity using probiotics have gained extensive attention because of their potential ability to regulate gut microbiota. Probiotics have been shown to increase the abundance of beneficial bacteria that can produce beneficial metabolites such as short-chain fatty acids, further promoting the oxidation of fatty acids and regulating appetite (8). Cheng Kong et al. found that the encapsulated probiotics’ preparation containing an equal ratio of *Lactobacillus* acidophilus, *Bifidobacterium longum*, and *Enterococcus faecalis* at a daily dose of 2 × 10^7^ colony-forming unit (CFU) could reshape high fat diet (HFD)-impacted gut microbiota in obese mice (9). It was reported that *Lactobacillus gasseri* SBT2055 could significantly reduce adipose tissue weight and adipocyte size by inhibiting dietary fat absorption in rats (10). *Lactobacillus reuteri* was also found to suppress hepatic fat deposits in HFD-induced obese mice by promoting the secretion of interleukin-22 (11). Moreover, probiotics were demonstrated to alleviate obesity-related endotoxemia. For instance, *Lactobacillus plantarum* LP104 could reduce the level of LPS in serum of HFD-fed mice (12). Currently, there are many studies about reversing obesity through probiotics or probiotic-contained foods. However, research regarding interventions for preventing obesity before the occurrence of obesity and obesity- related damages is relatively rare.

*Bifidobacterium animalis* subsp. *lactis* MN-Gup (MN-Gup, CGMCC No. 15578) is an aerospace mutation strain screened from the mutation strains of *Bifidobacterium animalis strain* BB-11 (CGMCC No. 14056) carried by Shenzhou-11 re-entry spacecraft, and it has been found to have the capacity of regulating gut microbiota and gut homeostasis (13, 14). In a previous study, we found that the intervention of fermented milk containing MN-Gup had a good performance in the treatment of obesity in an obese model using 9 weeks of HFD-fed rats (15). Nevertheless, whether MN-Gup works on preventing obesity is still unknown. In this study, C57BL/6J mice were administered with MN-Gup and fermented milk containing MN-Gup at the same time with HFD to investigate the protective effects of MN-Gup against HFD-induced obesity and endotoxemia. Additionally, the potentially protective mechanism was elucidated by gut microbiota analysis. This work will provide a more comprehensive understanding of the functions of MN-Gup on obesity, which helps to explore potential applications of MN-Gup.

## Materials and methods

### Animals and treatments

Male C57BL/6J mice (8 weeks old) were purchased from Charles River Laboratories (Beijing, China) and housed in an SPF-grade laboratory animal facility in Pony Testing International Group Co., LTD (Beijing, China). The study was approved by the institutional animal ethics committee (Approval Number: PO-NY-2019-FL-19). After 1 week of the acclimatization period, mice were randomly divided into seven groups with six mice in each group (Table 1). The low and high doses of MN-Gup were chosen based on the previously published study (13). Fermented milk was prepared using a commonly used procedure for conventional production of fermented milk according to the previous study (15), and the main ingredients of fermented milk were provided by Mengniu Hi-tech Dairy Product Beijing Co., Ltd (Beijing, China). Briefly, fermented milk was prepared using lactic acid bacteria starter cultures (*Streptococcus thermophilus* and *Lactobacillus bulgaricus*), and then low and high doses of MN-Gup were respectively added to prepare the test fermented milk containing probiotics. Erythritol was used as a calorie-free sweetener, and fermented milk was identical in energy (78 kcal), protein (3.0 g), fat (3.5 g), carbohydrate (5.3 g), Na (80 mg), and Ca (60 mg) content per 100 g. The test fermented milk was kept in cold storage and delivered weekly. The mice of NC group were fed with a normal-chow diet, and other groups of mice were fed with HFD (60% fat, TROPHIC, Nantong, China). Two groups of HFD-fed mice were intragastrically administered with 0.2 ml of sterile saline suspended with low and high doses of MN-Gup powder, respectively. Three groups of HFD-fed mice were intragastrically administered with 0.2 ml of fermented milk containing low and high doses of MN-Gup or without MN-Gup, respectively. NC and HFD control groups were intragastrically administered with 0.2 ml of sterile saline every day. The body weight of mice was recorded once a week. After 8 weeks of interventions, body composition (including body weight, fat percentage, and lean meat percentage) and magnetic resonance imaging (MRI) of mice were measured by a Small Animal Body Composition Analyzer (MesoQMR, Suzhou, China) on the second day after the last gavage. Then, mice were anesthetized with 1% pentobarbital sodium and their blood samples were collected to prepare serum. After 8 weeks of interventions, mice feces were collected before euthanasia on the second day after the last gavage.

**TABLE 1:** Experimental groups and diet of mice.

Group	Diet	MN-Gup content
Negative control (NC)	Normal-chow diet	–
High fat diet (HFD)	High fat diet	–
Low dose of MN-Gup powder (L-MG)	High fat diet	2 × 10^9^ CFU/kg b.w.
High dose of MN-Gup powder (H-MG)	High fat diet	1 × 10^10^ CFU/kg b.w.
Low dose of MN-Gup in fermented milk (L-MG-FM)	High fat diet	2 × 10^9^ CFU/kg b.w.
High dose of MN-Gup in fermented milk (H-MG-FM)	High fat diet	1 × 10^10^ CFU/kg b.w.
Fermented milk (FM)	High fat diet	–

### Measurement of blood lipid profile

Total cholesterol (TC), triglycerides (TG), low-density lipoprotein cholesterol (LDL-C), and high-density lipoprotein cholesterol (HDL-C) were measured using ELISA kits (Elabscience, Beijing, China), according to the manufacturer’s instructions. x

### Measurement of lipopolysaccharide in serum

Lipopolysaccharide was measured using ELISA kits (Elabscience, Beijing, China) according to the manufacturer’s instructions.

### Gut microbiota analysis

Gut microbiota analysis of fresh feces was completed by Shanghai Majorbio Bio-pharm Technology Co., Ltd (Shanghai, China). Microbial DNA was extracted from fecal samples using the MOBIO Power Fecal DNA Isolation Kit (Qiagen, Germany) according to the manufacturer’s instructions. The hypervariable region V3–V4 of the bacterial 16S rRNA gene was amplified with primer pairs 338F (5′-ACTCCTACGGGAGGCAGCAG-3′) and 806R (5′-GGACTACHVGGGTWTCTAAT-3′). The PCR product was extracted from 2% agarose gel and purified using the AxyPrep DNA Gel Extraction Kit (Axygen Biosciences, Union City, CA, USA) according to the manufacturer’s instructions and quantified using Quantus™ Fluorometer (Promega, USA). Purified amplicons were pooled in equimolar and paired-end sequenced on an Illumina MiSeq PE300 platform (Illumina, San Diego, CA, USA) (16). The sequencing data have been deposited in the National Center for Biotechnology Information (NCBI) and the project accession number is PRJNA867026. Operational taxonomic unit (OTU) cluster and taxonomy analysis were carried out using UPARSE and RDP Classifier algorithms, respectively. A principal coordinates analysis (PCoA) based on the unweighted-unifrac distances of the OTUs was performed to investigate the β diversity. Differentially abundant taxa were performed using linear discriminant analysis effect size (LEfSe).

### Statistical analysis

Data were expressed as means ± standard deviation (SD), and were statistically analyzed by one-way ANOVA followed by Duncan’s *post-hoc* test (SPSS, IBM, Armonk, New York, NY, USA).

## Results

### Protective effects of MN-Gup against high fat diet-induced obesity in mice

**FIGURE 1:**
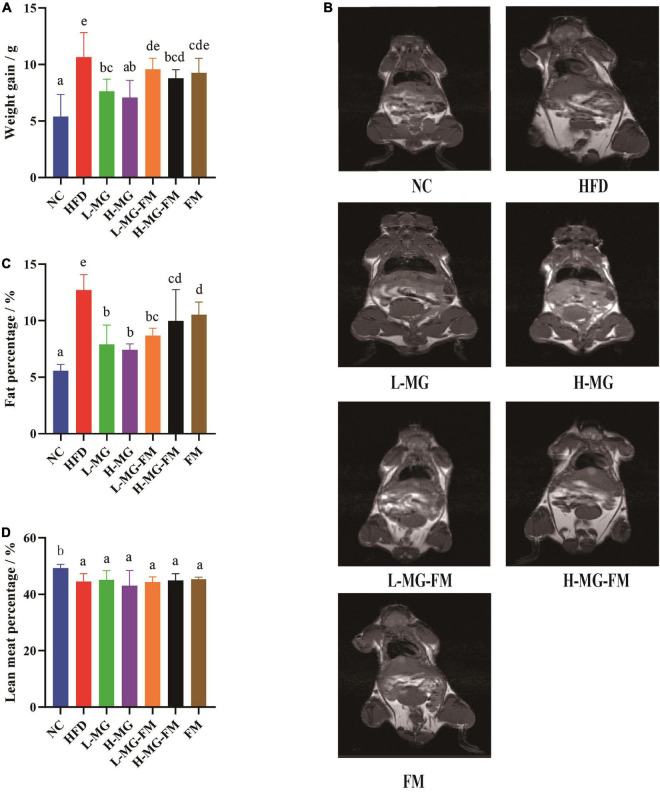
Protective effects of MN-Gup against HFD-induced obesity in mice. **(A)** Body weight gain of mice; **(B)** typical nuclear magnetic resonance images showing fat mass; **(C)** fat percentage in mice; **(D)** lean meat percentage in mice. Different letters indicate significant differences in mice (*p* < 0.05, *n* = 6).

As shown in Table 2, there was no significant difference in the initial body weight of all groups, and the mice with HFD gained more weight than the mice with a normal-chow diet (NC group). After 8 weeks, more than 15% body weight gain was observed in HFD group relative to NC group (Table 2), and the total body weight gain of HFD group was significantly more than NC group (*p* < 0.05, Figure 1A), indicating 8 weeks of HFD led to obesity in mice. However, MN-Gup effectively suppressed body weight gain of HFD-treated mice, and in particular, H-MG caused the fewest body weight gain (Figure 1A). Although the body weight of mice in fermented milk groups (L-MG-FM, H-MG-FM, and FM) was significantly lower than that of HFD group (Table 2), the weight gains of L-MG-FM and FM had no significant difference compared to that of HFD (Figure 1A). Notably, H-MG-FM significantly suppressed body weight gain of mice compared to HFD (*p* < 0.05, Figure 1A). Additionally, the nuclear magnetic resonance images showed that the fat mass of HFD group was apparently increased compared to NC group, which was reduced by interventions (Figure 1B). All interventions significantly reduced the fat percentages of mice induced by HFD (*p* < 0.05, Figure 1C), but did not significantly alter the lean meat percentages (Figure 1D). The fat percentages of L-MG and H-MG were lower than those of L-MG-FM and H-MG-FM (Figure 1C), suggesting that MN-Gup powder was more effective in preventing body fat accumulation than MN-Gup-containing fermented milk. Compared to FM, L-MG-FM, and H-MG-FM induced lower fat percentages (Figure 1C), which revealed that the supplement of MN-Gup could improve the anti-obesity effect of fermented milk. These results showed that MN-Gup could protect mice against HFD-induced obesity and its applications in fermented milk also had the same potential effect.

**TABLE 2:** Trends of body weight in mice.

Week	Body weight/g						
	NC	HFD	L-MG	H-MG	L-MG-FM	H-MG-FM	FM
0	22.96 ± 0.40^a^	22.98 ± 0.53^a^	22.66 ± 0.92^a^	22.91 ± 0.74^a^	22.43 ± 0.51^a^	22.58 ± 0.28^a^	22.51 ± 0.40^a^
1	24.45 ± 0.57^a^	25.88 ± 0.63^b^	24.30 ± 1.18^a^	24.33 ± 0.79^a^	24.43 ± 0.88^a^	24.45 ± 0.74^a^	24.63 ± 0.67^a^
2	25.33 ± 0.82^a^	27.30 ± 0.68^b^	25.56 ± 1.12^a^	25.21 ± 0.78^a^	26.20 ± 0.99^a^	25.66 ± 0.90^a^	25.91 ± 0.75^a^
3	26.15 ± 1.04^a^	28.83 ± 0.71^b^	26.58 ± 1.00^a^	26.35 ± 1.00^a^	27.15 ± 1.20^a^	26.95 ± 1.26^a^	27.11 ± 0.81^a^
4	26.75 ± 1.15^a^	30.31 ± 1.33^c^	27.43 ± 1.16^ab^	27.55 ± 1.15^ab^	28.31 ± 0.94^b^	27.91 ± 1.19^ab^	28.05 ± 0.61^ab^
5	27.13 ± 1.02^a^	31.08 ± 1.49^c^	28.61 ± 0.42^b^	28.28 ± 1.09^ab^	29.25 ± 1.11^b^	29.28 ± 1.24^b^	28.48 ± 0.73^b^
6	27.53 ± 1.20^a^	31.40 ± 2.18^c^	28.33 ± 0.40^ab^	28.78 ± 1.12^ab^	29.68 ± 2.38^bc^	29.41 ± 1.29^ab^	29.33 ± 0.61^ab^
7	28.05 ± 1.42^a^	32.80 ± 2.23^c^	29.33 ± 0.20^ab^	29.31 ± 0.88^ab^	30.33 ± 1.16^b^	30.40 ± 0.92^b^	30.45 ± 0.99^b^
8	28.38 ± 1.61^a^	33.98 ± 2.60^d^	30.30 ± 0.50^bc^	30.00 ± 1.00^b^	32.01 ± 0.89^c^	31.38 ± 0.66^bc^	31.78 ± 1.02^c^

Different letters indicate significant differences, *p* < 0.05, *n* = 6.

### Protective effects of MN-Gup against high fat diet-induced dyslipidemia in mice

**FIGURE 2:**
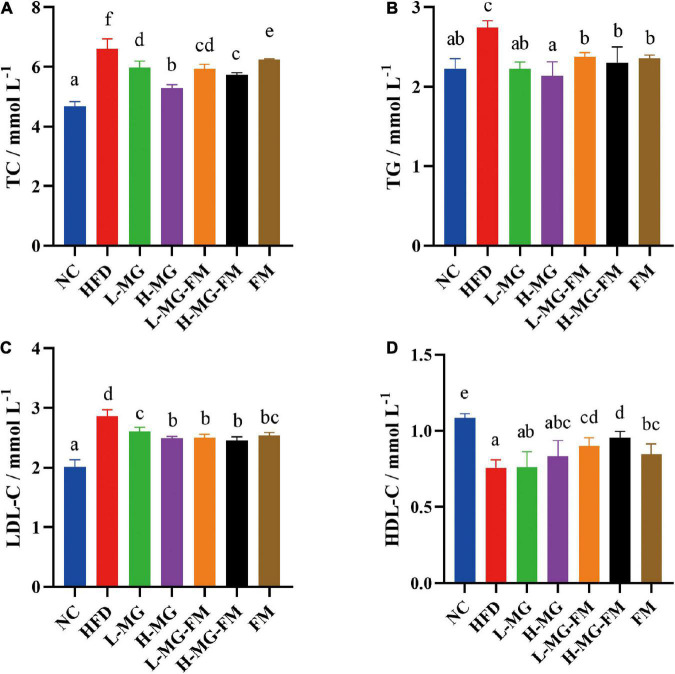
Serum lipid profiles in mice. The concentrations of **(A)** total cholesterol (TC), **(B)** triglyceride (TG), **(C)** low-density lipoprotein cholesterol (LDL–C), and **(D)** high-density lipoprotein cholesterol (HDL–C) were measured in serum. Different letters indicate significant differences (*p* < 0.05, *n* = 6).

High fat diet has been reported to induce dyslipidemia. Compared to NC, HFD feeding caused significant increases in the levels of serum TC, TG, and LDL-C and a significant decrease in the level of HDL-C (*p* < 0.05, Figures 2A–D). In contrast, interventions with MN-Gup powder or fermented milk containing MN-Gup could significantly attenuate HFD-elevated TC, TG, and LDL-C levels, and H-MG showed the best protection against elevated blood lipids (*p* < 0.05, Figures 2A–C). Surprisingly, interventions with MN-Gup powder (L-MG and H-MG) did not significantly change the HFD-decreased HDL-C, while fermented milk with or without MN-Gup significantly enhanced HDL-C levels, (*p* < 0.05, Figure 2D). Both L-MG-FM and H-MG-FM resulted in higher HDL-C levels than FM, suggesting that the supplement of MN-Gup could promote fermented milk to regulate HDL-C. These results showed that MN-Gup could protect against HFD-induced dyslipidemia.

### Protective effects of MN-Gup against high fat diet-induced endotoxemia in mice

**FIGURE 3:**
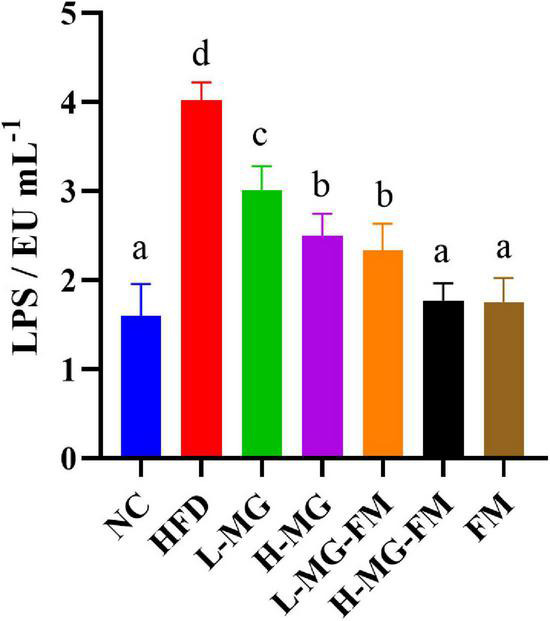
Lipopolysaccharide (LPS) content in serum of mice. Different letters indicate significant differences in mice (*p* < 0.05, n = 6).

The level of LPS in serum is commonly used to characterize endotoxemia. As shown in Figure 3, the LPS level in HFD group was significantly higher than that in NC group (*p* < 0.05), and all interventions could significantly attenuate HFD-elevated LPS levels (*p* < 0.05). The level of LPS in H-MG was significantly lower than that in L-MG, which was similarly observed between L-MG-FM and H-MG-FM (*p* < 0.05), suggesting that the modulatory effect of MN-Gup on LPS was dose-dependent. Furthermore, the LPS content in H-MG-FM and FM groups was significantly lower than that in the other intervention groups (*p* < 0.05), suggesting that fermented milk may have a better performance in protecting against HFD-induced endotoxemia.

### MN-Gup regulates gut microbiota in high fat diet-fed mice

**FIGURE 4:**
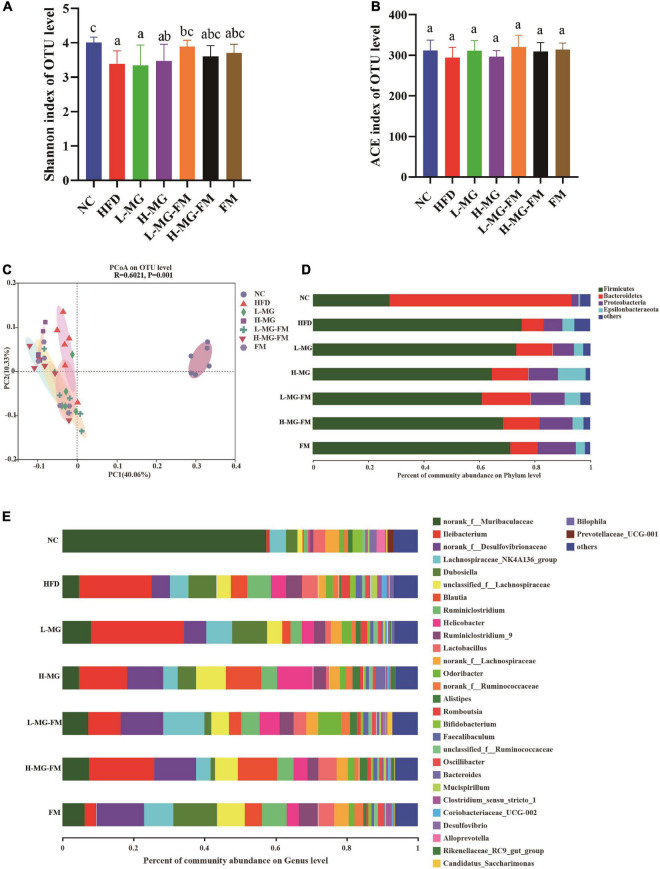
Effects of MN-Gup and MN-Gup-containing fermented milk on gut microbiota under HFD (*n* = 6). **(A,B)** The Shannon and abundance-based coverage estimators (ACE) indexes of bacterial α diversity. Different letters indicate significant differences in mice (*p* < 0.05). **(C)** PCoA based on bacterial operational taxonomic units (OTUs) using unweighted-unifrac calculation. ANOSIM was used to analyze the similarity analysis, and R value > 0 indicates that the difference between groups is greater than the difference between groups, and *p* < 0.05 indicates significant differences. **(D)** The average relative abundance of bacteria at the phylum level. **(E)** The average relative abundance of bacteria at the genus level.

There has been accumulating evidence for the crucial role of gut microbiota in HFD-induced obesity. To understand the changes in gut microbiota and the potential regulation of MN-Gup, we performed 16S rDNA gene sequencing of feces in mice. As shown in Figures 4A,B, α diversity of the community, including Shannon and abundance-based coverage estimators (ACE) indexes, was assessed. HFD caused a significant decrease in the Shannon index, indicating that HFD could reduce microbial diversity (Figure 4A). MN-Gup powder did not apparently affect the Shannon index, but fermented milk especially L-MG-FM increased the Shannon index (Figure 4A). There was no significant difference in the ACE index among groups, which suggested that HFD did not influence the species richness of gut microbiota (Figure 4B). Beta diversity based on PCoA showed that the cluster of HFD mice was clearly separated from the cluster of NC mice, suggesting that HFD feeding could apparently change gut microbiota composition of mice (Figure 4C). The interventions could change HFD-induced gut microbiota composition to various degrees, and in particular, H-MG resulted in the most distinct clustering from HFD group (Figure 4C). The taxonomic profiling also revealed the differences among groups. At the level of phylum, the dominant microbes could be classified as four phyla, including Firmicutes, *Bacteroidetes*, *Proteobacteria*, and *Epiasilonbacteraeota* (Figure 4D). Compared to NC, HFD had higher average relative abundances of Firmicutes, *Proteobacteria* and *Epiasilonbacteraeota* and a lower relative abundance of *Bacteroidetes* (Figure 4D). In contrast, the average relative abundance of Firmicutes was lowered and the average relative abundance of *Bacteroidetes* was enhanced by all interventions (Figure 4D). At the level of genus, the dominant bacterial including *norank_f__Muribaculaceae*, *Ileibacterium*, were altered by HFD compared to NC group (Figure 4E).

**FIGURE 5:**
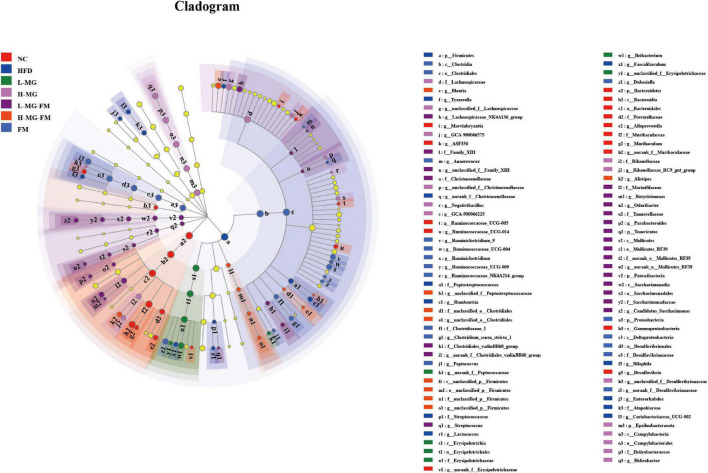
LEfSe analysis. Cladogram showing the evolutionary relationship between taxa that are differentially abundant between groups, where the threshold of the log (linear discriminant analysis) LDA score was set as 2 (*n* = 6).

LEfSe is a useful tool for discovering high-dimensional biomarkers and characteristics between groups in two or more groups of samples (17). As shown in Figure 5, *Bacteroidetes* (phylum), *Muribaculaceae* (family), and *Alloprevotella* (genus) were significantly enriched in NC group, which was consistent with the taxonomic profiling. The most abundant bacterial taxa in response to HFD included *Firmicutes* (phylum), *Coriobacteriaceae_UCG-002* (genus), *Romboutsia* (genus), *Tyzzerella* (genus), *Bilophila* (genus), *Enterorhabdus* (genus), etc. *Ileibacterium* (genus) enriched in L-MG. *Rikenellaceae* (family) and *Lachnospiraceae* (family) showed higher abundances in H-MG (Figure 5). The phyla including Patescibacteria and Tenericutes as well as Marinifilaceae (family) and *Odoribacter* (genus) were enriched in L-MG-FM group, *Blautia* (genus) was significantly enriched in H-MG-FM, and the genera including *Ruminiclostridium* (genus) and *Rumminococcaceae* (genus) were enriched in FM group (Figure 5). The above results suggested that MN-Gup and MN-Gup-contained fermented milk could regulate HFD-altered gut microbiota.

### Correlation between gut microbiota and obesity or endotoxemia

**FIGURE 6:**
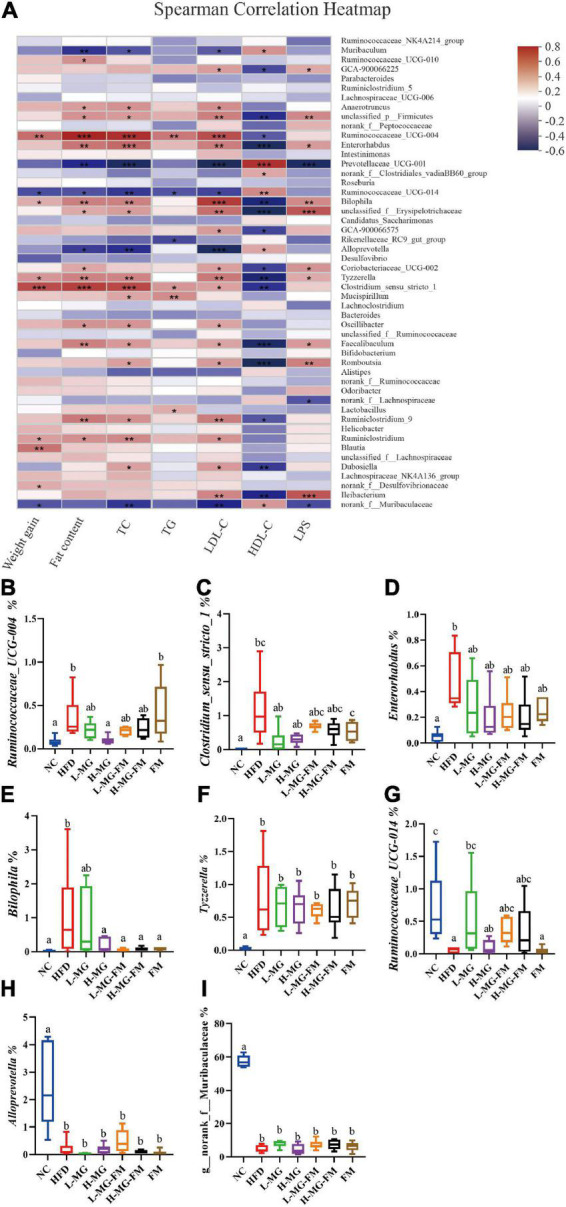
Correlation between gut microbiota and obesity or endotoxemia (*n* = 6). **(A)** Heat map of Spearman’s correlation between bacterial genera and obesity-related phenotypes or LPS level (Different colors indicate the correlation between bacterial genera and phenotypes, and red indicates positive correlation and blue indicates negative correlation. *0.01 < *p* ≤ 0.05, **0.001 < *p* ≤ 0.01, ***p ≤ 0.001). **(B–I)** The relative abundances of bacterial genera significantly correlated with obesity-related phenotypes or LPS level.

To further identify the relationship between the MN-Gup-regulated gut microbiota and obesity or endotoxemia, Spearman’s correlation analysis was conducted. The heatmap in Figure 6A showed the top 50 bacterial genera for total abundances associated with body weight gain, fat content, TC, TG, LDL-C, HDL-C, and LPS levels. There were 9, 18, 20, 6, 23, 21, and 13 genera significantly correlated with body weight gain, fat content, the levels of TC, TG, LDL-C, HDL-C, and LPS, respectively. Notably, *Ruminococcaceae_UCG-004*, *Enterorhabdus*, *Bilophila*, *Tyzzerella*, and *Clostridium_sensu_stricto_1* were positively correlated with body weight gain, fat content, the TC, TG, LDL-C, and LPS levels and negatively correlated with HDL-C level (Figure 6A). As shown in Figures 6B–F, *Ruminococcaceae_UCG-004*, *Clostridium_sensu_stricto_1*, *Enterorhabdus*, *Bilophila*, and *Tyzzerella* were significantly increased in HFD (*p* < 0.05), but reduced by interventions, especially H-MG. In contrast, *Ruminococcaceae_UCG-014*, *Alloprevotella*, and *Norank_f__Muribaculaceae* were negatively correlated with body weight gain, fat content, TC, TG, LDL-C, and LPS levels and positively correlated with HDL-C level (Figure 6A), which were all significantly reduced in HFD (*p* < 0.05, Figures 6G–I). However, interventions did not show significant effects on the relative abundances of *Ruminococcaceae_UCG-014*, *Alloprevotella*, and *Norank_f__Muribaculaceae* (Figures 6G–I). It could be inferred that MN-Gup may alleviate obesity and endotoxemia by regulating the gut microbiota associated with obesity and LPS.

## Discussion

Many studies have shown that probiotics including *Lactobacillus* and *Bifidobacterium* have significant effects on the treatment of obesity and related complications (18, 19). *Bifidobacterium longum* APC1472 supplementation was reported to have the potential of reducing obesity (20), and *Bifidobacterium pseudocatenulatum CECT 7765* administration could ameliorate obesity-related neuroendocrine alterations in mice (21). *Bifidobacterium animalis subsp. lactis BB-12* could also ameliorate obesity by regulating gut microbiota in rats (22). Additionally, *Lactobacillus plantarum* A29 reduced the fat mass and serum lipid profiles via down-regulating adipogenic gene expression in adipocytes and altering gut microbiota in mice (23). Given the wide applications of probiotics in fermented milk, fermented milk containing probiotics also has attracted attention to alleviate obesity. For example, fermented milk containing *Lactobacillus gasseri SBT2055* could significantly reduce visceral fat, subcutaneous fat, and body weight gain in obese subjects (24), and fermented milk containing active *L. reuteri NCIMB 30242* could reduce the levels of LDL-C and TC in patients with hyperlipidemia (25). In the previous study, fermented milk containing MN-Gup has been found to have the potential to treat obesity in rats (15), but the role of MN-Gup in preventing obesity has never been known. Previous studies mostly focused on the effects of probiotics on pre-existing obesity using therapy models, in which the mice or rats were firstly treated to reach a state of obesity, and then interventions of probiotics were performed. However, it may make more sense to prevent obesity before it happens than to treat an individual having been obese, which may prevent many obesity-related diseases from occurring. Therefore, the preventive effects of MN-Gup and fermented milk containing MN-Gup on HFD-induced obesity were investigated in this study. Additionally, the current study investigated different objects and phenotypes by comparison with the previous study (15). The effects of various doses of MN-Cup were evaluated and the differences between MN-Gup powder and the corresponding MN-Gup-contained fermented milk were compared in the current study, while the previous study focused on the comparison between MN-Gup and its combination with prebiotics in fermented milk. In addition to obesity-related phenotypes, the current study evaluated the preventive effect of MN-Gup on endotoxemia, which was not involved in the previous study.

Gut microbiota plays a crucial role in maintaining host health and regulating homeostasis, and changes in gut microbiota composition can affect host health and disease (26–28). There is some evidence indicating that the gut microbiota responds quickly and accurately to diet and affects various metabolic conditions including obesity (29, 30). The current study showed that HFD caused an imbalance of gut microbiota, which was reshaped by MN-Gup and fermented milk containing MN-Gup. As shown in Figure 4E, compared to control group (NC), the relative abundance of *Bifidobacterium* was reduced in HFD group, but the proportion of *Bifidobacterium* was not apparently elevated by the MN-Gup intervention. *Bifidobacterium* contains a wide variety of species, and MN-Gup was just a specie of *Bifidobacterium*. This result suggested that HFD had an apparent influence on total *bifidobacterium*, which may be difficult to be reversed by MN-Gup interventions. According to the results of LEfSe analysis (Figure 5), Firmicutes, *Coriobacteriaceae_UCG-002*, *Romboutsia*, *Tyzzerella*, *Bilophila*, and *Enterorhabdus* were significantly enriched in HFD group. Previous research has shown that *Firmicutes*, *Coriobacteriaceae_UCG-002*, and *Romboutsia* were positively correlated with the increase of fat and blood lipids (31–33). *Tyzzerella* has been proven to be related to the consumption of sugar and carbohydrate (34). *Bilophila* was reported to be related to fat growth (35) and *Enterorhabdus* was associated with diabetes or other metabolic diseases (36). The characteristic genus of L-MG group was *Ileibacterium* and this is consistent with previous research results (37). *Lachnospiraceae* is a characteristic family of H-MG group, which was believed to produce short-chain fatty acids to suppress obesity (38, 39). As for L-MG-FM group, *Marinifilaceae* was demonstrated to alleviate lipid metabolism disorder, and the characteristic *Odoribacter* and *Butyricimonas* are bacteria with the capability of producing butyrate (40–42). In the characteristic strain of H-MG-FM, *Blautia* was found to significantly inhibit the growth of visceral fat area (43). *Ruminiclostridium* and *Rumminococcaceae* boosted in FM, however, the association between these two bacteria and obesity is rarely reported. More importantly, bacterial genera correlated with obesity or endotoxemia, and their changes were analyzed to elucidate the potential mechanism of MN-Gup in preventing HFD-induced obesity and endotoxemia.

The body weight and body fat of mice significantly increased with HFD, which was consistent with many studies (31, 44, 45). L-MG and H-MG had the best effect on suppressing body weight gain, while the body weight gain in H-MG-FM was significantly lower than L-MG-FM and FM (Figure 1A). As expected, HFD also significantly enhanced fat percentage and reduced lean meat percentage (Figures 1C,D). It has been reported that the fat accumulation is closely linked to the elevated *Firmicutes* (31), and a higher proportion of *Firmicutes* was observed in HFD compared to NC (Figure 4D). *Firmicutes* proportion was decreased by all interventions (Figure 4D), which may be associated with the decreased fat percentage (Figure 1C). However, all interventions could not significantly elevate lean meat percentage compared to HFD group (Figure 1D), suggesting that HFD-induced muscle loss was difficult to improve (46).

Dyslipidemia is a common complication of obesity due to excessive accumulation of glycerol and fatty acids in the blood (47). The levels of TC, TG, and LDL-C were significantly increased by HFD, which were reduced by all interventions (Figures 2A–C). Some studies have shown that *Clostridium_sensu_stricto_1*, *Enterorhabdus*, and *Bilophila* were associated with elevated blood lipids (32, 36, 48), whose proportions were significantly higher in HFD group (Figure 6). In contrast, interventions reduce the proportions of these bacteria (Figure 6), which may be involved in the decreased TC, TG and LDL-C levels. Interestingly, both fermented milk with or without MN-Gup could significantly enhance HFD-reduced HDL-C, but L-MG and H-MG had no significant effects (Figure 2D). The improvement of HDL-C in L-MG-FM and H-MG-FM was better than that in FM, suggesting the supplement of MN-Gup could promote fermented milk to regulate blood lipids.

Recent evidence in rodents and humans suggests that gut-derived endotoxemia may play an important role in obesity and obesity-related metabolic disorders (49). Endotoxemia, namely excessive accumulation of LPS in blood, is attributed to gut barrier impairment and gut microbiota dysbiosis. It was demonstrated that HFD induced elevations in LPS (50, 51). The level of LPS was significantly increased in HFD, which was significantly reduced by all interventions (Figure 3). Previous studies have shown that probiotics such as *Bifidobacterium animalis* ssp *lactis 420* could prevent intestinal barrier damage and endotoxemia by inhibiting harmful bacteria and promoting the proportion of *Lactobacillus* (52). It was reported that *Bilophila* could promote abnormal intestinal barrier function (48), and H-MG, L-MG-FM, H-MG-FM, and FM could significantly reduce HFD-elevated *Bilophila* (Figure 6E). In addition, the positively LPS-correlated bacterial genera, including *Ruminococcaceae_UCG-014*, *norank_f_Muribaculaceae* were also decreased by MN-Gup and fermented milk containing MN-Gup. These results indicated that MN-Gup may alleviate endotoxemia by regulating the gut microbiota.

Although fermented milk itself contains *Streptococcus thermophilus* and *Lactobacillus bulgaricus* (conventional starter cultures) that may have interactions with MN-Gup on regulating gut microbiota, the effects of MN-Gup could be proved by comparing the fermented milk group (FM) and fermented milk with MN-Gup groups (L-MG-FM or H-MG-FM). There were some limitations in the current study. The overall effect of fermented milk containing MN-Gup on preventing obesity was better than fermented milk without MN-Gup but seemed to be weaker than MN-Gup powder regarding body weight and fat gain, where the mechanism was not explored in this study. Moreover, how MN-Gup-regulated gut microbiota contributed to the prevention of HFD-induced obesity and endotoxemia was not fully elucidated, which need to be studied in the future.

## Conclusion

In summary, this study demonstrated that *Bifidobacterium animalis* subsp. *lactis* MN-Gup (MN-Gup) significantly attenuated body weight gain, fat percentage, dyslipidemia, and endotoxemia with HFD in mice. Notably, high dose of MN-Gup (H-MG) had the best performance on preventing obesity, and fermented milk with MN-Gup had better preventive effects on fat percentage and dyslipidemia than fermented milk without MN-Gup. HFD-affected gut microbiota and obesity- or endotoxemia-correlated bacteria could be regulated by MN-Gup and MN-Gup-containing fermented milk, which may contribute to the prevention of obesity. These results suggest the promise of MN-Gup as a functional ingredient for preventing obesity and improving public health.

## Data availability statement

The datasets presented in this study can be found in online repositories. The names of the repository/repositories and accession number(s) can be found below: National Center for Biotechnology Information (NCBI), and the project accession number is PRJNA867026.

## Ethics statement

This animal study was reviewed and approved by the Institutional Animal Ethics Committee of Pony Testing International Group Co., Ltd.

## Author contributions

XN, NZ, XK, and JZ conceived and designed the experiments and wrote and revised the manuscript. XN and NZ performed the experiments. RW, QZ, and JH assisted in data analysis. SL, NL, and ES provided technical support. All authors contributed to the article and approved the submitted version.
